# Mechanisms of cancer pain

**DOI:** 10.3389/fpain.2022.1030899

**Published:** 2023-01-04

**Authors:** Rayan Haroun, John N Wood, Shafaq Sikandar

**Affiliations:** ^1^ Division of Medicine, Wolfson Institute of Biomedical Research, University College London, London, United Kingdom; ^2^ William Harvey Research Institute, Barts and the London School of Medicine and Dentistry, Queen Mary University of London, London, United Kingdom

**Keywords:** pain, dorsal root ganglia, cancer-induced bone pain, chemotherapy associated pain, neuropathy, cancer, sensitisation

## Abstract

Personalised and targeted interventions have revolutionised cancer treatment and dramatically improved survival rates in recent decades. Nonetheless, effective pain management remains a problem for patients diagnosed with cancer, who continue to suffer from the painful side effects of cancer itself, as well as treatments for the disease. This problem of cancer pain will continue to grow with an ageing population and the rapid advent of more effective therapeutics to treat the disease. Current pain management guidelines from the World Health Organisation are generalised for different pain severities, but fail to address the heterogeneity of mechanisms in patients with varying cancer types, stages of disease and treatment plans. Pain is the most common complaint leading to emergency unit visits by patients with cancer and over one-third of patients that have been diagnosed with cancer will experience under-treated pain. This review summarises preclinical models of cancer pain states, with a particular focus on cancer-induced bone pain and chemotherapy-associated pain. We provide an overview of how preclinical models can recapitulate aspects of pain and sensory dysfunction that is observed in patients with persistent cancer-induced bone pain or neuropathic pain following chemotherapy. Peripheral and central nervous system mechanisms of cancer pain are discussed, along with key cellular and molecular mediators that have been highlighted in animal models of cancer pain. These include interactions between neuronal cells, cancer cells and non-neuronal cells in the tumour microenvironment. Therapeutic targets beyond opioid-based management are reviewed for the treatment of cancer pain.

## The problem of cancer pain

Cancer survival rates have dramatically increased since the turn of the century with the advent of targeted and improved treatment strategies. On the other hand, the annual number of cancer cases worldwide is estimated to rise from 14 million in 2012 to 22 million by 2032, leaving behind the growing problem of effective pain management for side effects of cancer and its treatments. Almost all patients diagnosed with cancer endure chronic pain because of surgery, treatments or side effects like a pathological fracture. Between 30% and 50% of patients receiving curative-intent therapy and 75%–90% of patients with advanced disease endure chronic pain that is strong enough to require opioid therapy (1, 2). More than one-third of patients in disease remission will continue to report pain after curative treatment (3). Cancer-related pain often consists of background pain with acute exacerbations, peaking several times a day that can be either spontaneous or evoked in areas of sensory abnormality. Spontaneous pain may be ongoing (either at a constant or fluctuating pain intensity), or it can be dominated by a juxtaposition of pain paroxysms of short duration interspersed by pain-free intervals or a less intense background pain. Paraesthesia and dysaesthesia are also frequently reported.

The World Health Organization (WHO) Analgesic Ladder is frequently the first step in a paradigm for guiding clinicians to manage pain in a systemic manner, where the selection of pharmacological analgesic treatments is based on the degree of pain. Over-the-counter analgesics comprise Step 1, “weak” opioids (such as codeine) are escalated in Step 2, followed by the use of “strong” opioids in Step 3 to treat moderate-to-severe pain. Clinical professionals are urged to take non-pharmacologic pain management methods in Step 4 into account (4). Non-opioid analgesics include acetaminophen (5) and non-steroidal anti-inflammatory drugs (NSAIDs) (6). The advantage of NSAID/opioid combination therapy is a reduction in overall opioid prescriptions, but data on its efficacy is conflicting (6–8). Adjuvant therapies include anti-depressants [like duloxetine (9) and anticonvulsants like gabapentin and pregabalin (10)], which are first or second-line analgesics for other chronic pain conditions, including painful neuropathies (11). Adjuvant analgesics, integrative therapeutic options [such as acupuncture (12)], as well as interventions [e.g., nerve block (13) and epidural or intrathecal analgesics (7)] can be considered at any stage of pain management even though they are not listed on the WHO ladder.

There remains a continuous debate regarding the suitability of these generalised WHO recommendations to adequately manage pain in a heterogeneous group of patients that have a cancer diagnosis. A meta-analysis of WHO Cancer Pain Relief guideline outcomes indicates that adequate analgesia is achieved in a range of as little as 20% but up to 100% of patients (14). A contentious point has been raised regarding the prescription of weak Step 2 opioids before starting morphine for managing moderate pain; patients with cancer suffering from moderate pain have a higher probability of responding to low-morphine doses than they are to codeine (15). Furthermore, new research indicates that potent analgesics may be more effective if given earlier in the course of the disease prior to a critical transition point when the plasticity of the nociceptive system may become resistant to conventional pharmacological treatment (16).

The need to modify current pain management protocols in patients with cancer is indicated by reports that pain is the most common complaint leading to emergency unit visits by patients with cancer (17–19). Despite well-established findings demonstrating that the experience of pain can impact long-term clinical outcomes, patients with cancer frequently receive insufficient pain management (20). Studies investigating the frequency and effectiveness of pain control suggest areas for improvement, e.g., a systematic analysis reported that despite a 25% drop in under-treated cancer pain between 2007 and 2013, over one-third of patients with cancer still experience under-treated pain (21). Pain has a demonstrable impact on the quality of life of patients with cancer, and the lack of adequate pain management among this growing population underlines the need for novel and targeted approaches to treat pain arising from cancer or its treatment. This review summarises the use of animal models of cancer pain, with a focus on cancer-induced bone pain (CIBP) and chemotherapy associated pain. Peripheral and central nervous system mechanisms involved in cancer pain are also discussed, followed by potential targets for pain relief.

## Animal models of cancer pain

Here we describe animal models of cancer pain, including method of induction and pain related outcomes.

### Animal models of cancer-induced bone pain (CIBP)

Early models of CIBP relied on the administration of cancer cells into the left ventricle of mice, followed by migration of cancer cells in the general circulation to develop metastases at different tissue sites, including bone marrow (22). The main advantage of models relying on systemic administration of cancer cells models is the replication of the clinical course of disease due to the fact that bone tumours usually develop as metastases rather than as primary tumours. However, significant disadvantages of such models include the poor predictability of metastatic sites and size, leading to insufficient reproducibility of disease progression across a cohort of animals. Another strategy is to directly administer cancer cells into the intramedullary space of a bone (e.g., the femur) in rodents (23). The site of the injection of the cancer cells into the bone is then sealed to restrict tumour growth in the intramedullary space. In comparison to systemic administration of cancer cells, CIBP models that involve direct injection in the bone facilitate the evaluation of tumour growth over time, radiological imaging, observation of bone degradation, examination of histopathologic changes, site-specific behavioural testing, as well as evaluation of neurochemical changes that take place at the tumour site, in the dorsal root ganglion (DRG) and the central nervous system (CNS) (24). Mouse models typically involve the implantation of tumour cells into the femur, and rat models are typically created *via* percutaneous injection of cancer cells into the tibia (25). The cell lines used will vary across species and studies, e.g., for B6C3-Fe-a/a and C3H/HeJ mice, fibrosarcoma cells have been reported to be administered in the femur (26, 27), humerus (28) and calcaneus bones (29). Lewis lung carcinoma cells are administered into the intramedullary space of the femur in C57BL/6 mice (30). In rats, MRMT1 mammary gland carcinoma cells are administered into the tibias of female Sprague–Dawley rats (31), and MDA-MB231 human breast cancer cells are injected into femoral arteries of nude rats (32) as well as R3327 prostate cancer cells (33). Cancer cells that have metastasised to the bone can be classified as either osteosclerotic or osteolytic based on x-ray images (34). Osteosclerotic cancers are distinguished by increased bone deposition through increased osteoblast activity. Prostate cancer is the protype for osteosclerotic bone metasases, whereas breast cancer is the protype for osteolytic bone metastases because breast cancer cells potentiate osteoclast-induced bone degradation (34, 35).

Rodents exhibit pain-like behaviours within 2–3 weeks of intrafemoral inoculation with cancer cells in a dose-dependent manner (30, 36, 37). Non-evoked pain behaviours include reduced use of the affected limb, which can be quantified using a limb score, as well as a reduction in weight borne on the affected limb when tested by an incapacitance tester (30, 36–38). Loss of bone mineral density or osteolysis can also be reliably detected with the progression of tumour growth (30). The ability to reproduce a chronic pain phenotype, as well as the cancer-induced bone remodelling, is critical to understanding the structural and neuronal mechanisms underlying CIBP (39, 40). One major shortcoming of animal models is the challenge in predicting breakthrough pain, which is an important clinical feature of CIBP. Assessment of breakthrough pain would require continuous monitoring of animals, e.g., with the use of ultrasound vocalisation techniques, but has yet to be an established feature of rodent CIBP models. See Table 1 for a summary of different animal models of CIBP.

**Table 1:** Animal models used to study CIBP and targets for analgesia.

Murine model and strain	Method of induction of cancer pain model	Assays to assess pain behaviour	Analgesics tested (and reference)
CIBP in C3H/HeNCrl mice	Injection of osteolytic NCTC 2472 fibrosarcoma cells into the femur	Spontaneous lifting Limb-use during forced ambulation on rotarod	Fentanyl (0.025–0.64 mg/kg, s.c) Sufentanil (0.005–0.04 mg/kg, s.c) Morphine (2.5–40 mg/kg, s.c) (41)
CIBP in athymic nude mice	Injection of Canine prostate carcinoma cells into the intramedullary space of the femur	Time spent in spontaneous guarding and flinching Thermal and mechanical sensitivity.	Antibodies against the nerve growth factor (NGF) (10 mg/kg, i.p.) Morphine sulfate (10 or 30 mg/kg, s.c) (42)
CIBP in C3H/HeJ mice	Injection of 2472 osteolytic sarcoma cells into the intramedullary space of the femur	Spontaneous flinching Flinching following normally non-noxious palpation Nocifensive behaviour scoring	Osteoprotegerin (daily s.c. injection of 50 μl containing 2.25 mg/ml Osteoprotegerin) (43)
CIBP in C3H/HeJ mice	Injection of 2472 osteolytic sarcoma cells into the intramedullary space of the femur	Mechanical hypersensitivity (von Frey) Spontaneous guarding	Morphine (15 mg/kg s.c.) (44)
CIBP in C3H/HeJ mice	Injection of NCTC 2472 tumour line into the intramedullary space of the femur	52°C hot plate Guarding and flinching behaviours measurement Limb-use score Tactile hypersensitivity (von Frey)	Morphine (1, 3, 10, 20 mg/kg/day, s.c) (45)
CIBP in C3H/HeJ mice	Injection of NCTC 2472 tumour line into the intramedullary space of the femur	Spontaneous flinches Limb-use scoring Activity-related guarding	Osteoprotegerin (5 mg/kg s.c. daily from day 12 after sarcoma inoculation till day 21) (46)
CIBP in C3H/HeJ mice	Injection of NCTC 2472 tumour line into the intramedullary space of the femur	Guarding and flinching. Limb-use and rotarod tests. Touch-evoked pain (von Frey) Palpation-induced guarding Palpation-induced flinching.	Morphine sulfate (0.3–300 mg/kg i.p.) (47)
CIBP in C3H/HeJ mice	Injection of NCTC 2472 tumour line into the intramedullary space of the femur	Spontaneous flinching and guarding Palpation-evoked guarding and flinching Limb-use scoring	ABT-627 (a selective antagonist of endothelin receptor A) (10 mg/kg, i.p.) A-192621 (a selective antagonist of endothelin receptor B) (30 mg/kg, i.p.) (48)
CIBP in C3H/HeJ mice or B6C3-Fe-a/a mice	Injection of NCTC 2472 tumour line into the intramedullary space of the femur	Light palpation of the tumour followed by scoring of the nocifensive response	N/A (26)
CIBP in C3H/HeNCrl mice	Injection of NCTC 2472 tumour line into the intramedullary space of the femur	Spontaneous lifting behaviour Palpation-evoked lifting behaviour Limb-use during forced ambulation	Morphine sulphate (15 mg/kg, i.p.) (49)
CIBP in C3H/HeNCr mice	Injection of NCTC 2472 fibrosarcoma cells into and around the calcaneus	Mechanical hypersensitivity (von Frey) Heat hyperalgesia (Hargreaves’ test)	N/A (50)
CIBP in C3H/He mice	Injection of clone 2472 fibrosarcoma cells into the mouse calcaneus bone	Mechanical hyperalgesia (von Frey filaments) Cold hyperalgesia (3 ± 1°C cold plate)	N/A (29)
CIBP in C3H/He mice	Injection of clone 2472 fibrosarcoma cells into the mouse calcaneus bone	Mechanical sensitivity (von Frey test)	TNFR:Fc (i.pl. (0.001 pg to 1.0 ng/20 μl) or i.p. (100–500 μg/mouse) or intratumour (0 μg/20 μl/mouse) (51)
CIBP in C3H/HeJ mice	Injection of clone 2472 fibrosarcoma cells into and around the calcaneous bone	Mechanical sensitivity (von Frey) Cold hyperalgesia (2.5°C ± 1°C cold plate)	N/A (52)
CIBP in C3H/He mice	Injection of NCTC clone 2472 cells into the humerus	Measurement of forelimb grip force	WIN55,212-2 (1–30 mg/kg, i.p.) (53)
CIBP in C3H/HeJ mice	Injection of NCTC clone 2472 cells into the cavity of the humerus	Ambulation on a rotarod Assessing the grip force	Radiation Morphine-hydrochloride (5 mg/kg, i.p.) Ketorolac (30 mg/kg, i.p.) (54)
CIBP in C3H/He or B6C3/F1 mice	Implantation of NCTC 2472 sarcoma cells into the humeri (C3H/He) or melanoma cells (B6C3/F1 mice)	Grip force test Cutaneous hypersensitivity (von Frey)	Morphine (3–100 mg/kg, i.p.) (28)
CIBP in C3HHeJ mice	Injection of 2472 sarcoma cells into the intramedullary space of the femur	Palpation-induced flinches Limb-use score Limb-use while ambulating	Selective COX-2 inhibitor (chronic MF tricyclic, ∼30 mg/kg/day, p.o.) or (acute NS-398 (100 mg/kg, p.o) (55).
CIBP in immunocompromised C3H-SCID mice	Injecting 2472 sarcoma, B16 melanoma or C26 colon adenocarcinoma into the femur	Spontaneous guarding Guarding during forced ambulation on a Rotorod Guarding after innocuous palpation of tumour-bearing limb	N/A (38)
CIBP in Swiss CD1 mice	Injection of XC Rous sarcoma-virus-transformed rat fibroblast cells	Thermal hyperalgesia (55 ± 1°C hot plate) Mechanical hyperalgesia (Randall and Selitto) Capsaicin-Induced Licking	Morphine (1–10 mg/kg, i.p.) BQ-123 (10 nmol, i.pl.) BQ-788 (10 nmol, i.pl) (56)
CIBP in C3H/HeJ mice	Injection of NCTC 2472 cells intratibially	Thermal hyperalgesia (55 ± 1°C hot plate) Spontaneous pain [modified Dubuisson and Dennis method (57)]	Morphine (15 mg/kg, i.p) (58)
CIBP in C3H/HeJ mice	Injection of NCTC 2472 cells into the medullary cavity of the tibia	Thermal hyperalgesia (55 ± 1°C hot plate)	Morphine (10 nmol, s.c. over the tibial tumour mass) Loperamide (146 nmol, s.c. over the tibial tumour mass) (59)
CIBP in C3H/HeJ mice	Injection of SCC-7, squamous cell carcinoma cells into the thigh	Heat (radiant heat) Mechanical sensitivity (von Frey) Spontaneous pain (including lifting, licking, and flinching)	Capsazepine (10 or 30 mg/kg, i.p.) (60)
CIBP in C3H/HeJ mice	Injection of hepatocarcinoma cells, HCa-1 into the thigh or the dorsum of the foot	Mechanical sensitivity (von Frey test) Cold allodynia (acetone test) Heat hyperalgesia (radiant heat)	N/A (61)
CIBP in C3H/HeJ mice	Injection of hepatocarcinoma, hca-1 cells into the periosteal membrane of the foot dorsum	Mechanical sensitivity (von Frey) Cold sensitivity (acetone) Heat hyperalgesia (radiant heat)	Radiation (62)
CIBP in C3H/HeJ mice	Injection of hepatocellular carcinomahca-1 cells into the periosteal membrane of the foot dorsum	Mechanical sensitivity (von Frey test) Cold sensitivity (acetone) Heat hyperalgesia (radiant heat)	Radiation (63)
CIBP in C57BL/6 mice	The injection of Lewis lung carcinoma cells into the intramedullary space of the femur	Limb-use score Weight bearing	Nav1.7 knockout from DRG and sympathetic neurons. Ablation of Nav1.8-positive neurons (64)
CIBP in C57BL/6 mice	The injection of Lewis lung carcinoma cells into the intramedullary space of the femur	Limb-use scoring Weight-bearing Rotarod Mechanical sensitivity (von Frey) 50°C hot plate test Dry ice test	N/A (37)
CIBP in Sprague–Dawley rats	Injection of MRMT-1 rat mammary gland carcinoma cells into the medullary space of the proximal tibia	Cold bath test (4°C) Hot plate test (38°C for allodynia and 52°C for hyperalgesia) Mechanical hyperalgesia (Randall Selitto test) Thermal hyperalgesia (infrared beam) Tactile allodynia (von Frey test) Weight bearing test	Morphine (3 or 5 mg/kg, s.c.) Lacosamide (3, 10, 20, 30 or 40 mg/kg, i.p.) (65).
CIBP in Wistar rats	Implantation of Walker 256 carcinoma cells into the plantar surface of rat hind paw	Mechanical Hyperalgesia (Randall–Selitto Test) Mechanical sensitivity (von Frey) Spontaneous pain behaviour (lifting and licking of the affected paw)	Indomethacin (4 mg/kg, i.v.) Morphine (10 mg/kg, s.c.) (66)
CIBP in Sprague–Dawley rats	Injection of MRMT-1 tumour cells into the tibia	Weight-bearing Mechanical sensitivity (von Frey)	Lumiracoxib (10 and 30 mg/kg, p.o.) Valdecoxib (30 mg/kg, p.o.) (67)
CIBP in Wistar rats	Injection of 256 rat mammary gland carcinoma cells into the tibia	Limb-use score Mechanical sensitivity (von Frey) Hargreaves test Weight-bearing test	N/A (68)
CIBP in Sprague–Dawley rats	Injection of MRMT-1 rat mammary gland carcinoma cells into the tibia	Mechanical sensitivity (von Frey) Mechanical hyperalgesia (paw pressure) Overall daily activity	N/A (31)
CIBP in Sprague–Dawley rats	Injection of MRMT-1 rat mammary gland carcinoma cells into the tibia	Heat hyperalgesia (radiant heat) Grip force test Foot stamp test	Zoledronic acid (250 µg/kg/day, s.c.) (69)
CIBP in Sprague Dawley rats	Injection of MRMT-1 cell line into the tibia	Ambulatory-evoked pain (rotarod test) Mechanical sensitivity (von Frey) Cold allodynia (acetone)	Gabapentin (30 mg/kg, s.c. chronic) or (10, 30 or 100 mg/kg, s.c. acute) (70)
CIBP in Copenhagen rats	Injection of AT-3.1 prostate cancer cells in the tibia	Thermal hyperalgesia (radiant heat) Mechanical hyperalgesia (paw pressure) Mechanical sensitivity (von Frey) Spontaneous flinching	Morphine (5 mg/kg or 10 mg/kg, s.c.) (71)

### Animal models of non-bone cancer pain

Besides the models of cancer pain arising from bone, other animal models of primary cancers originating from different organs have been described, such as pancreatic cancer, squamous cell carcinoma and neuroma. One mouse model of pancreatic cancer involves the elastase 1 promoter-driven expression of the simian virus 40 large T antigen (72). In this model, pathological effects of these precancerous cells can be observed after 6 weeks, such as elevation in microvasculature density, the number of nerve growth factor (NGF) expressing macrophages and increased density of sensory and sympathetic fibers innervating the pancreas. Although these aforementioned changes would be expected to lead to severe pain in somatic structures like the skin, no apparent changes in pain-related behaviours, like morphine-reversible severe hunching and vocalisation, are observed in this model of pancreatic cancer until an advanced stage of disease after 16 weeks (72). These findings imply that a stereotypical series of pathological changes are present in both humans and mice as pancreatic cancer progresses. Although weight loss typically corresponds to disease progression, there is often a considerable delay from the onset of tumour growth to behaviours suggestive of pancreatic cancer pain (72). Clinical presentation of back and abdominal pain in pancreatic cancer is commonly reported at an advanced stage of disease in more than 80% of cases (73), but the underlying causes of the discrepancy between neuronal innervation and symptomatic pain in pancreatic cancer remain to be investigated.

Another preclinical model of pancreatic cancer that involves the implantation of SW 1990 cells into the pancreas of female BALB/c-nu mice carries several advantages, including reproducible transcriptional changes at the level of the dorsal horn in both mice and humans with pancreatic cancer (74). Pain-like behaviours in pancreatic cancer-bearing mice can be assessed by stimulating the abdomen mechanically (by von Frey hairs) to assess mechanical withdrawal thresholds and score hunching behaviours, as well as electrophysiologically record visceromotor responses from the rectus abdominis (74). An animal model of squamous cell carcinoma involves the injection of cancer cells into the subperiosteal tissue of the lower gingiva, which leads to elevated levels of calcitonin gene-related peptide, Substance P, P2X3 receptors and TRPV1 channels in the trigeminal ganglia, coinciding with the development of mechanical hypersensitivity and thermal hyperalgesia (75). Finally, the tibial neuroma transposition model involves the ligation of the tibial nerve and placement above the lateral malleolus—while not perfectly representative of the natural clinical course of disease progression, this model can be useful for mechanistic investigations of pain due to tumour-induced nerve injury (76, 77).

Patients with head and neck cancer experience pain early in the disease process, and orofacial pain is often the presenting symptom for oral squamous cell carcinoma (SCC). Schmidt and collleagues have pioneered the development of mouse models of oral cancer pain, either through orthotopic oral tissue injection of human SCC to produce operant nocifensive behaviours measured using the Dolognawmeter (to measure gnaw time) (78). Other work has used administration of SCC cells to the hindpaw to investigate mechanisms of mechanical allodynia (79). See Table 2 for a summary of different animal models of non-bone cancer pain.

**Table 2:** Some of the animal models used to study pain in non-bone cancer and targets for analgesia.

Murine model and strain	Method of induction of cancer pain model	Assays to assess pain behaviour	Analgesics tested (and reference)
A mouse model of pancreatic cancer (transgenic mouse)	Transgenic mouse expressing the simian virus 40 large T antigen under control of the rat elastase-1 promoter	Hunching behaviour Palpation-evoked vocalisation	Morphine sulfate (10 mg/kg, s.c) (72)
A rat model of Squamous cell carcinoma in Fisher rats	The inoculation of SCC-158 cells into the subperiosteal tissue of the rats on the lateral side of the lower gingiva	Mechanical sensitivity: von Frey (whisker-pad skin and submandibular skin) and dynamic plantar aesthesiometer (paw). Thermal sensitivity: 55 ± 0.5°C hotplate (whisker-pad skin and submandibular skin) and radiant heat (paw).	N/A (75)
A rat model of neuroma-induced pain in Sprague–Dawley rats	A neuroma is permitted to develop after the tibial nerve is ligated and positioned just superior to the lateral malleolus.	Neuroma tenderness (von Frey). Hindpaw mechanical hyperalgesia (von Frey).	Lidocaine injection (100 μl injection of 1% lidocaine with epinephrine, s.c.) Proximal tibial nerve transection (76)
Mouse models of pain in oral squamous cell carcinoma (SCC) in BALB/c nude mice	50 μl of human SCC (cells for chronic pain, supernatant for acute pain) injected into the left lateral tongue.	Dolognawmeter (operant measure)	Soybean trypsin inhibitor (20 μg intratumour) (78)

### Animal models of cancer related neuropathic pain

Neuropathic pain is a key feature in some animal models of pain upon either cancer invasion or treatment with chemotherapy (80, 81). For the former, cancer cells are implanted in close proximity to nerves, e.g., sciatic nerve, and standardised behavioural assays measure changes in hindlimb sensitivity (81). Animal models of cancer invasion typically cause more profound neuronal damage than classic rodent models of neuropathic pain, such as the chronic constriction injury model, and the degree and effects of nerve compression after cancer appear mechanistically distinct (81).

Neurotoxicity is one of the key treatment-limiting side effects of chemotherapy, which can manifest as painful peripheral neuropathy or chemotherapy-induced neuropathic pain. Across classes of chemotherapeutic agents, the extent of polyneuropathy depends on the dose and length of treatment but is generally associated with an acute phase of allodynia and pricking dysaesthesia affecting the hands and feet in patients. Acute or persistent neuropathic pain can be reproduced by systemic administration of chemotherapeutic agents in rodents. Some studies also report the development of motor neuropathy, gait abnormalities and impaired rotarod performance (82), which mimic aspects of sensory impairment besides hypersensitivity that develops in chemotherapy-induced peripheral neuropathy (83). For example, daily intravenous administration of vincristine, a vinca alkaloid, elicits dose-dependent mechanical and cold (but not heat) hypersensitivity within 2 days (84–86) that is attenuated 2 weeks after cessation of treatment (84). Paclitaxel is a commonly used taxane in rodent models of chemotherapy-induced peripheral neuropathy to produce both thermal and mechanical hypersensitivity (87, 88). A recent study on the responses of 10 mouse strains to paclitaxel administration revealed that nearly all strains experienced mechanical allodynia and cold allodynia, including DBA/2J and C57BL/6J mice (89). Cisplatin is a platinum derivative that also results in numbness, tingling, as well as painful neuropathy in humans (90, 91). In rats, typically, three doses totaling 15 mg/kg are administered to produce mechanical allodynia and hyperalgesia lasting up to 15 days (92). The fast onset of neuropathic symptoms is a major benefit of this model (92). Additionally, while motor nerve conduction velocity is unaltered in electrophysiological studies, a considerable drop in sensory nerve conduction velocity can be detected (93). Lastly, preclinical studies using oxaliplatin-induced neuropathic pain have highlighted key mechanisms associated with cold allodynia that occur with chemotherapy administration. Mice administered 80 µg intraplantar oxaliplatin show a significant reduction in pain thresholds assessed using the cold plate test. Symptoms of cold allodynia become evident as early as 3 h after oxaliplatin administration (94). An important advantage of this model is its rapid onset resembling the rapid cold allodynia seen in patients with cancer treated using oxaliplatin (95). See Table 3 for a summary of different animal models of chemotherapy associated pain.

**Table 3:** Some of the animal models used to study chemotherapy-associated pain and targets for analgesia.

Murine model and strain	Method of induction of cancer pain model	Assays to assess pain behaviour	Analgesics tested (and reference)
Vincristine-induced neuropathy in Sprague-Dawley rats	Vincristine (100 μg/kg into a tail vein) administration on daily basis for 2 weeks.	Heat hyperalgesia (radiant heat) Mechanical hyperalgesia (Randall Selitto paw-withdrawal) Mechanical sensitivity (von Frey) Rotarod test for potential motor effects	DAMGO [(D-Ala2, N-MePhe4, Gly-ol)-enkephalin] (1 µg, i.d) (84)
Vincristine-induced neuropathy in Sprague-Dawley rats	Injection of Vincristine (50 or 75 μg/kg) into the tail vein of rats daily for 10 consecutive days.	Mechanical hyperalgesia (Randall-Selitto test) to measure vocalisation threshold Von Frey test. Heat hyperalgesia (radiant heat) Tail immersion test (42°C water bath)	N/A (96)
Vincristine-induced neuropathy in Sprague–Dawley rats	Injection of vincristine at a single dose of 50, 100 and 200 μg/kg, i.v)	Mechanical hyperalgesia [increasing mechanical force (20–160 g)] Mechanical sensitivity (von Frey) Rotarod test	Inhibitors of protein kinase Cε, A, G P42/p44-mitogen activated protein kinase inhibition Nitric oxide synthase inhibitors (all compounds i.d. 1 μg/paw) (97)
Vincristine-induced neuropathy in Sprague–Dawley rats	Vincristine is applied through jugular vein pump at a dose of 30 μg/kg/day	Thermal hyperalgesia (radiant heat) Mechanical hyperalgesia (Randall-Selitto test) Paw immersion in (4.5°C) water Mechanical sensitivity (von Frey test)	Dextromethorphan hydrobromide monohydrate (p.o.) Aspirin (p.o.) Ibuprofen (p.o.) Acetaminophen (p.o.) Carbamazepine (p.o.) Clonidine hydrochloride (i.p.) Morphine hemi[sulfate pentahydrate] (i.p.) Desipramine hydrochloride (i.p.) Gabapentin (p.o) Celecoxib (i.p.) Lamotrigine (p.o) (86)
Vincristine-induced neuropathic pain in	Vincristine was infused at a dose of 1–100 µg/kg/day for 14 days	Mechanical sensitivity (von Frey) Heat withdrawal threshold using lightbulb heat source	Morphine sulfate (5 mg/kg, i.p.) Lidocaine hydrochloride (45 mg/kg, i.p.) Mexiletine (30 mg/kg, i.p.) Tetrodotoxin (8 μg/kg, i.p.) Pregabalin (80 mg/kg, i.p.) (85)
Paclitaxel-induced neuropathy in CD1 mice	21.6 mg/kg of paclitaxel (i.p.) for 6 consecutive days	Tail flick temperature	NGF (10 µg/gm, i.p., for 7 days) (98)
Paclitaxel-induced neuropathy in ddy mice	Paclitaxel injection (4 mg/kg, i.p.)	N/A	Gabapentin (3–30 mg/kg, i.p.) (99)
Paclitaxel-induced neuropathic pain in 129P3, A, AKR, C3H/He, C57BL/6, C57BL/10, CBA, DBA/2, RIIIS, and SM (all “J” substrains) mice	Several paclitaxel injections on days (1, 3, 5, and 7, i.p.). The total paclitaxel dose was 4 mg/kg	Mechanical sensitivity (von Frey test) Thermal hyperalgesia (Hargreaves’ test) Cold allodynia (acetone)	N/A (89)
Paclitaxel-induced neuropathy in Dark Agouti rats	Injection of paclitaxel at a dose of 9 mg/kg (i.p.) twice a week. Dosing continued until 20% weight loss is achieved.	Tail flick test Rotarod test Gait disturbance	Oral sodium glutamate (∼500 mg/kg/day, p.o.) (82)
Paclitaxel-induced peripheral neuropathy in Sprague–Dawley rats	0.1, 0.5 or 1 mg/kg taxol (i.p, once a day for 2 weeks)	Mechanical hyperalgesia (Randall–Selitto test in the paw) Mechanical sensitivity (von Frey) Thermal sensitivity (Hargreaves’ test)	Pkcɛ-I inhibitor (1 μg/paw, i.d.) PKA inhibitor (1 μg/paw, i.d.) (100)
Cisplatin-induced neuropathy in Dark Agouti rats	Injection of cisplatin at a dose of 2 mg/kg (i.p.) twice a week. Dosing continued until 20% weight loss is achieved.	Tail flick test Rotarod test Gait disturbance	Oral sodium glutamate (∼500 mg/kg/day, p.o.) (82)
Cisplatin-induced neuropathy in Sprague-Dawley rats	Weekly injection of cisplatin (2 mg/kg, i.p.) for 10 weeks	Walking track	Decompressive surgery (101)
Cisplatin-induced neuropathy in Sprague-Dawley rats	Four to five cisplatin injections at 3 mg/kg at weekly intervals	Mechanical sensitivity (von Frey) Thermal sensitivity (Hargreaves’ test)	Morphine (0.3, 0.6 and 1 mg/kg s.c.) (102) Amitriptyline (30, 70 mg/kg i.p.) Meloxicam (5, 10 and 20 mg/kg i.p.)
Oxaliplatin-induced cold allodynia in albino Swiss mice	Singles injection of oxaliplatin (10 mg/kg, i.p.)	Cold plate test (1°C, 2.5°C and 4°C)	Amitriptyline (1, 2.5 and 10 mg/kg, i.p.) (103)
Oxaliplatin-induced cold allodynia in C57BL/6 background mice	Injection of 80 µg of oxaliplatin (i.pl.).	Cold plate test (5°C)	Deletion of Nav1.8 positive neurons (94)

## Mechanisms of cancer pain

### Cancer-induced bone pain (CIBP)

**Figure 1:**
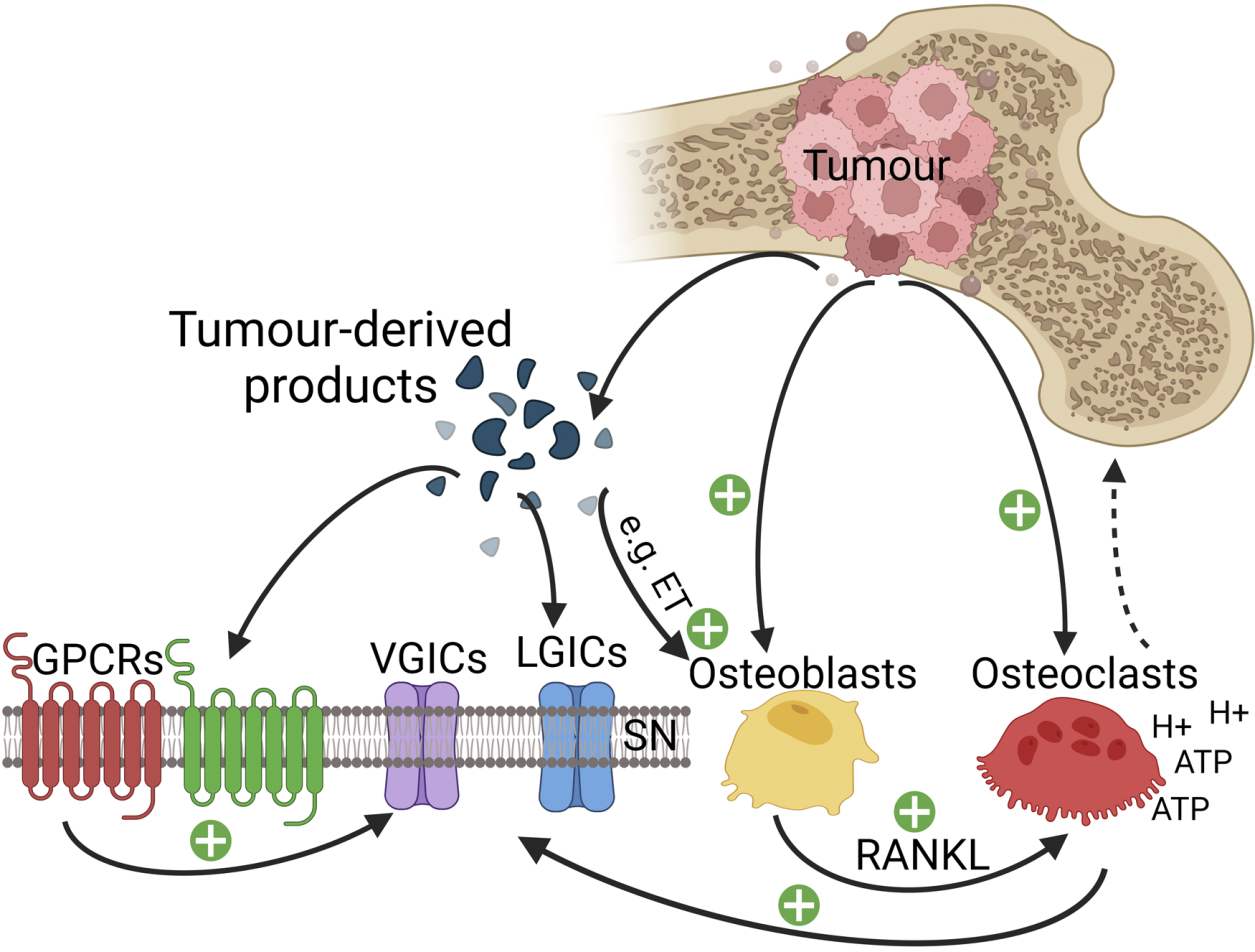
Cellular interactions in the bone microenvironment in CIBP. Tumour cells release endothelin (ET), which interacts with osteoblasts *via* their appropriate receptors to stimulate the proliferation of osteoblasts. Activated osteoblasts release receptor activator of nuclear factor-kappa-Β ligand (RANKL), which serves as a signal for osteoclast proliferation and maturation to enhance osteoclast-mediated bone matrix destruction. Osteoclasts generate adenosine triphosphate (ATP) and acidosis by releasing protons, resulting in the activation of various receptors and ligand-gated ion channels (LGICs) like P2X receptors, transient receptor potential V1 receptors and acid-sensing ion channels type 3 expressed on bone innervating sensory neurons. Tumour cells, stromal cells and activated immune cells release a variety of mediators (such as endothelin, the nerve growth factor, protons, and pro-inflammatory cytokines) that activate their respective receptors expressed on sensory neurons and thereby initiate the detection of noxious stimuli. GPCRs can sometimes indirectly sensitise various voltage-gated ion channels (VGICs) expressed on sensory neurons leading to a further potentiation of nociceptive signalling to the spinal cord. Osteolytic cancers (like breast cancer) activate osteoclasts, while osteosclerotic cancers (like prostate cancer) activate osteoblasts leading to a further potentiation of pain signal transmission.

Paget's “seed and soil” hypothesis of the late 19th century was seminal to our current understanding that certain tumours exhibit a predilection for metastasis to specific organs (104). Bone is the optimal “soil” for metastatic cells of primary tumours, most commonly from breast, prostate myeloma, thyroid, lung and bladder cancer. Post-mortem examinations revealed that the incidence of bone metastases is around 70% for patients with breast or prostate cancer and 36% for patients with lung cancer (105). Bone metastases are the most common cause of cancer-related pain (105, 106) and are associated with poor prognosis and survival (107). Metastasis to bone disrupts skeletal homeostasis by disturbing the balance between osteoblastic bone formation and osteoclast-mediated bone destruction (108). Fenestrations in bone marrow sinusoids allow easy trafficking of hematopoietic cells but can also be permissive to metastatic invasion (109). Receptor activator of nuclear factor kappa-Β ligand (RANKL) is a chemotactic factor that promotes bone metastasis (110), and chemokine receptors such as CXCR4 (C-X chemokine receptor 4), which binds the survival chemokine stromal cell-derived factor 1 present on osteoblasts and bone lining cells, promote adhesion and metastasis within the bone microenvironment (111, 112). Within the bone, cancer cells directly compete with hematopoietic stem cells driving their terminal differentiation (113). In this microenvironment, bone-innervating neurons and cancer and tumour-associated stromal cells (including fibroblasts, endothelial cells, lymphocytes and bone marrow-derived cells, such as macrophages, neutrophils, mesenchymal stem cells and mast cells) can all contribute to CIBP through changes in bone homeostasis, structural and neurochemical reorganisation of sensory and sympathetic nerve fibres innervating bone, as well as the neurochemical reorganisation in the spinal cord. These processes highlight the importance of peripheral and central mechanisms driving the complex condition of CIBP that comprises inflammatory, neuropathic and cancer-specific mechanisms of nociceptive signalling. We discuss peripheral and central mechanisms below (see Figure 1).

Acidosis is thought to be a key mechanism driving CIBP and is largely driven by disseminated tumour cells that dysregulate bone remodelling through osteomimicry of osteoblast and osteoclasts (114–116). The disruption of skeletal homeostasis and acidic bone microenvironment can contribute to peripheral sensitisation through the activation of acid-sensing ion channels (ASICs) and transient receptor potential (TRP) channels. In the Warburg effect, cancer cells tend to undergo anaerobic respiration even in the abundance of oxygen, unlike normal cells that mainly undergo aerobic respiration in the abundance of oxygen and shift to anaerobic respiration when there is a shortage of oxygen supply (117). Among the products of anaerobic respiration is lactic acid (at physiological pH, lactic acid deprotonates to form lactate and protons), which causes acidosis. Cancer-induced acidosis is enhanced in bone cancers because the bone is a hypoxic tissue (118). In addition, tumour cells activate osteoclasts, which in turn cause bone degradation by releasing protons to solubilise the mineralised bone matrix (119). Upon the physiological activation of osteoclasts, the release of acids by osteoclasts during bone resorption is restricted to the bone matrix due to the tightly sealed sac called the resorption lacuna (120). Typically in bone cancer the number and activity of osteoclasts increase dramatically, thereby disrupting this tight regulation and resulting in the leakage of protons that can amplify acidity levels of the bone marrow. The bone marrow itself is richly innervated with nociceptors expressing ASICs and TRP channels—especially TRPV1—that are important sites of neuronal activation by protons (69, 121–124).

In addition to acidosis, several mediators released by cancer cells and their associated stromal cells contribute to CIBP. These include NGF and interleukin (IL)-1β (125), which can contribute to CIBP both directly and indirectly. For instance, IL-1β enhances the expression of cyclooxygenase (COX)-2 by macrophages, in turn amplifying prostaglandin synthesis. These prostaglandins can sensitise the primary afferent neurons by binding the prostanoid receptors expressed on terminals of bone innervating neurons (126, 127). Similarly, the production of NGF by cancer cells in the bone (e.g., derived from primary tumours of the breast and prostate) can lead to sensitisation of sensory neurons directly by increasing the expression ion channels linked to pain signal transduction and transmission. These include the TRPV1 channel, which is of particular interest in CIBP given the strong acidosis that characterises this painful condition (128). Moreover, in the spontaneous osteosarcoma canine model, TRPV1 blockade attenuates hypersensitivity suggesting it may be a potential analgesic target candidate in CIBP (122). NGF can also enhance the production and release of TNF-α, IL-6, IL-1β and prostaglandin (PG)E_2_ by macrophages (129). TNF-α is known to exert pro-nociceptive actions in animal models of chronic pain and can is targeted for pain relief in rheumatic disease (130). The peripheral pro-nociceptive actions of TNF-α are evident from the observation that intraplantar, intradermal, endoneurial or intramuscular administration of TNF-α induces thermal hyperalgesia and mechanical allodynia (131–135). In addition, TNF-α affects several ion channels like TRPV1, sodium and potassium channels (136–138) and results in the spontaneous firing of primary sensory neurons (139, 140). In preclinical models of CIBP, the administration of etanercept [a humanised soluble recombinant TNF receptor fusion protein (141)] was found to attenuate the thermal and mechanical allodynia in bone cancer-bearing mice (142). Additionally, blockade of the interaction between NGF and its receptor Tropomyosin receptor kinase A (TrkA) reduces pain-like behaviour in a mouse model of CIBP, with analgesic effects that are superior to morphine (143). Furthermore, several factors at the site of bone metastasis, such as reactive oxygen species (ROS), immune cell infiltration and activation, as well as tumour-induced cytotoxicity, result in the production of adenosine triphosphate (ATP) following the death of bone marrow cells. When ATP is released, it binds its receptor P2X3 on sensory neurons leading to their activation and subsequent sensitization (125). The blockade of P2X3 receptors can attenuate pain behaviours in a rat model of CIBP (144).

The neuropathic pain component of CIBP can be driven by increased intraosseous pressure, nerve sprouting and direct nerve injury. In humans, increased innervation density is also observed at sites of active bone remodelling, supporting the importance of neural regulation of skeletal remodelling and pain (145). Bone cancer pain, similar to intraosseous engorgement syndrome, produces intraosseous pressure within the bone microenvironment, which can sensitise primary afferents through activation of mechanoreceptors (146) and mechanotransducing osteocytes (147). Moreover, CIBP is characterised by the sprouting of sensory and sympathetic fibres, which has been shown to be driven by an NGF-dependent process. The requirement of NGF-TrkA signalling in bone innervating neurons for endochondral ossification and vascularisation, as well as bone formation upon mechanical loading, is well established (148, 149). In preclinical models of CIBP, prophylactic antibody-based blocking of NGF prevents ectopic sprouting and neuroma formation (150). The ablation of capsaicin-sensitive sensory neurons also results in loss of bone mineral density in adult rats (151). At the level of the peripheral somata, biomarkers that are indicative of nerve injury can also be detected, including cyclic activating transcription factor 3 (ATF3) expression. Spinal cord compression occurs in about 5% of patients with metastatic cancer, mainly presenting as back pain (152), and in animal models, spinal cord compression readily triggers mechanical hyperalgesia in the fore and hind limbs (153). Macrophage infiltration into peripheral sensory ganglia is also commonly observed in nerve injury models and studies highlight the importance of this neuro-immune interface in the development of CIBP (154).

At the level of the spinal cord, CIBP modulates synaptic plasticity between the peripheral neurons and second-order neurons, as observed through increased neuronal excitability measured with elevated expression of c-Fos, the internalisation of substance P, the rise in the expression of dynorphin (a pro-nociceptive opioid) and a significant activation and elevation in astrocytes and microglia (26, 155). In addition, CIBP has been shown to increase the proportion of the wide dynamic range to nociceptive-specific neurons in the superficial laminae of the dorsal horn in rats (156). It was reported that gabapentin can re-establish the typical ratio of wide dynamic range neurons to nociceptive-specific neurons, but long-term use of morphine maintains allodynia and fails to correct the pathophysiological phenotype of superficial dorsal horn neurons (157). Descending modulatory circuits also regulate spinal excitability in CIBP; 5-hydroxytryptamine type 3 (5-HT_3_) receptor antagonists can diminish the hyperexcitability of lamina I neurons in rodents with CIBP through the blockade of a descending serotonergic facilitatory drive (158). Other studies report an important role for amplified excitatory neurotransmission that may underlie enhanced spinal cord plasticity in CIBP; spinal glial cells secrete IL-1 that causes hyperalgesia by phosphorylating the NR1 subunit of N-methyl-D-aspartate receptor (NMDA) receptors (159). Moreover, spinal NR2B expression is increased in CIBP, and its specific inhibition prevents the induction of mechanical and thermal hypersensitivity (160). Due to hypertrophy of astrocytes and the subsequent decline in glutamate reuptake transporters, glutamate concentrations are also elevated in CIBP, which leads to excitotoxicity (161). Mice with CIBP also exhibit elevated amounts of dynorphin in the dorsal horn (26), which causes long-lasting pain through activation of NMDA receptors instead of opioid receptors (162).

### Chemotherapy-induced neuropathic pain

Chemotherapy-induced neuropathic pain has a detrimental impact on the quality of life of patients during and after chemotherapy treatment (163). Because chemotherapeutic agents do not normally cross the blood-brain barrier, neuropathies are restricted to peripheral sensory and/or motor neurons, leading to a reduction in two-point discrimination and proprioception, as well as cold allodynia, myalgia and pain in extremities (164). The severity and duration of neuropathy are determined by the class of the chemotherapeutic agent used, the total duration of administration, as well as the cumulative dose administered. If the cell body is spared and a sufficient time-period for recovery is allowed before subsequent drug administration, the peripheral nervous system regenerates rapidly after the cessation of chemotherapy treatment. Still, up to one-third of patients experience damage lasting more than 6 months after cessation of a chemotherapy course (165). How do chemotherapeutic agents predominantly cause sensory neuropathy while leaving the motor neurons unaffected, even though motor neurons have axons that are as long as those of sensory counterparts and have extensive microtubule networks? And why are adult postmitotic sensory neurons preferentially harmed by chemotherapy drugs that are meant to target fast-dividing tumour cells? One explanation for this latter susceptibility with vinca alkaloids and taxanes is that these classes of chemotherapy drugs interfere with the stability of microtubules that are necessary for the axonal transport of chemicals and growth factors required for normal nerve function in sensory neurons. Unfortunately, this rationale does not apply to platinum-based chemotherapy drugs that generate DNA adducts in the nucleus but also injure sensory neurons to trigger chemotherapy-induced neuropathic pain (163, 166).

Chemotherapeutic agents with a high incidence of chemotherapy-induced neuropathic pain include paclitaxel, oxaliplatin and vincristine. Studies from animals and humans indicate that these agents build up in peripheral sensory ganglia and, to a lesser extent, in peripheral nerves (167–169). The neurotoxic effects of these classes of chemotherapeutic agents lead to cell death and neuronal degeneration. Expression of ATF3 in DRG neurons is significantly increased with administration of paclitaxel, and the same neurons also exhibit a deposition of neurofilaments and a translocation of their nuclei towards the periphery. Dorsal roots of rats receiving paclitaxel also show substantial axonal degradation and hypomyelination (170). Moreover, exposure to NGF attenuates some neurotoxic effects triggered by paclitaxel in CD1 mice, including the release of neuropeptides in DRG neurons (98). Apoptosis of DRG neurons is thought to be partially responsible for chemotherapy-induced neuropathy, and a high dose of NGF can prevent DRG apoptosis induced by cisplatin (171, 172). Chemotherapeutic agents have also been shown to increase the generation of ROS, which impairs mitochondrial electron transport chains, and disrupts ATP synthesis in the DRG neurons (173–177). These findings support the role of mitochondrial stress pathways in the neurotoxic effects of chemotherapeutic agents.

Ion channels have also been linked to mechanisms of chemotherapy-induced neuropathic pain. Rat studies demonstrate that paclitaxel and vincristine administration enhance the expression of the calcium channel alpha_2_delta-1 subunit in the dorsal horn of the spinal cord. Moreover, ensuing mechanical hypersensitivity is susceptible to attenuation by repeated gabapentin-dosing, which is also associated with a decrease in the expression of the spinal alpha_2_delta-1 subunit (176). Previous work has also shown an important role for tetrodotoxin (TTX)-sensitive sodium channels in chemotherapy-associated pain in mice. Acute subcutaneous administration of TTX at doses as low as 1 or 3 mg/kg diminished mechanical allodynia in paclitaxel-treated mice, while cold allodynia and heat hyperalgesia were attenuated with higher doses (3 or 6 mg/kg) (175). The antagonist of TRPA1 channels HC-030031 partially attenuated paclitaxel-evoked mechanical allodynia (174), whilst co-administration of HC-030031 and HC-067047 (TRPV4 antagonist) reversed paclitaxel-induced mechanical allodynia entirely (173) in rodents. Furthermore, a key mechanism of cold allodynia induced by oxaliplatin administration is the activation of “silent cold-sensing neurons” that express the voltage-gated sodium channel Na_v_1.8 (94, 178). Oxaliplatin treatment in mice with diphtheria toxin-ablated Na_v_1.8-positive neurons failed to exhibit cold allodynia compared to wild-type controls (94).

Distinct effects of different classes of chemotherapeutic agents are less studied. However, it is known that paclitaxel and vincristine-induced neuropathy trigger significant inflammatory processes which are less evident with oxaliplatin-induced neuropathy, mainly arising from the enhanced release of proalgesic mediators such as TNF and IL-1β by activated microglia, astrocytes and satellite glial cells within the dorsal horn of the spinal cord (179). The mechanism of action of vinca alkaloids that involves the prevention of microtubule formation by binding tubulin has also been shown to affect micro-tubuli, causing oedema of axons in the peripheral nervous system (163). Paclitaxel administration is also associated with a significant increase in macrophage activation and augmented staining for glial fibrillary acidic protein (GFAP). Following paclitaxel administration, satellite cells are reported to be tightly packed in “Nodules of Nagoette” that serve as a “tombstone” for the DRG neuron whose cell body they formerly encircled (180).

### Mechanisms of pain in non-bone cancers

In pancreatic cancer, pain is the third most prevalent complaint among patients diagnosed with pancreatic cancer after weight reduction and jaundice (181). At the time of diagnosis, more than one-third of patients complain of abdominal discomfort and are already at an advanced stage of disease, but with disease progression, pain becomes severe in more than half of patients diagnosed with pancreatic cancer (182). Pain reports in recently diagnosed patients with pancreatic cancer can also predict survival and resectability, where preoperative pain is linked with a poor prognosis and a greater likelihood of recurrence (183). As a consequence of late diagnoses, the typical survival time of patients with pancreatic cancer ranges between 6 and 9 months, with the 5-year survival rate being less than 5% (184). Deciphering mechanisms that suppress nociceptive signalling in the early stages of pancreatic cancer and/or amplify nociceptive signalling in advanced disease is critical for improving the diagnosis, treatment and care of patients with pancreatic cancer.

Perineural invasion is a key feature of pancreatic cancer, which is enabled by the low resistance of the perineural space as well as the ideal milieu possessing chemoattractants and growth factors (such as transforming growth factor-alpha, epidermal growth factor receptor, and neural cell adhesion protein) to recruit cancer cells and promote their proliferation (185–187). A recent study showed that pancreatic cancer cells derived from patients had higher levels of TRPV1 gene expression with a downstream effect of the enhanced release of neuropeptides and augmented neurogenic inflammation (188). In mouse models of pancreatic cancer, extensive sprouting of sensory and sympathetic fibres is also detected (72) and partly attributed to the high levels of NGF released by the cancer cells and/or the inflammatory cells (187). Specifically, mice develop increased microvascular density, NGF-expressing macrophages and sensory and sympathetic innervation to the pancreas at 6 weeks following the induction of the pancreatic cancer model—all pathological events that are linked to precancerous cellular abnormalities (72, 189). Despite early changes in cell and tissue composition of the pancreatic microenvironment, pain-like behaviours are only detected after 16 weeks of age when pancreatic cancer is at an advanced stage, similar to clinical reports of pain in patients at advanced stages of the disease. Some studies suggest that regulation of nociceptive input by the descending opioidergic controls contributes to the slow evolution of tissue injury (190, 191) and that endogenous opioids may mask the full expression of pain at the early stages of disease (192). When naloxone or naltrexone were given subcutaneously to mice with early- and mid-stage pancreatic cancer, behaviours associated with pain were observed in these mice, while the healthy littermate controls failed to demonstrate pain behaviours (189). Unmasking of endogenous opioid regulation of pain behavior has also been observed in a rat model of prostate cancer induced bone pain, as well as a rat model of breast cancer induced bone pain (193, 194). A salient point is that this opioid-based un-masking of pain only occurs with opioid antagonists penetrating the blood-brain barrier, but not with peripherally-restricted opioid antagonists (192).

Visceral pain in pancreatic cancer can be caused by pancreatic neuropathy driven by cancer cells that infiltrate the perineurium of local intrapancreatic nerves (195). It is also thought that the initiation and maintenance of pain after pancreatic cancer is through neurogenic inflammation (187). In addition, several genes shown to be linked to pain are upregulated in the dorsal horn of the spinal cord in murine models of pancreatic cancer, such as *Ccl12*, *Pin1* and *Notum* (74). The palmitoleoyl-protein carboxylesterase encoded by *Notum* is implicated in the Wnt signalling-mediated initiation and maintenance of neuropathic pain (196). Moreover, mRNA levels of *Ccl12* increase by up to 35-fold in the prostate of mice with experimental autoimmune prostatitis (a model of chronic pelvic pain syndrome) (197).

In oral SCC, patients exhibit high levels of ET-1 in the cancer microenvironment, which has been shown to correlate with functional pain in response to mechanical stimulation (198, 199). Protein and mRNA levels of NGF are also significantly higher in patients with oral cancer and in oral SCC culture (200). Other studies have linked protease-activated receptor 2 (PAR2) to cancer pain (201). For instance, the supernatant of human oral SCC cells contains proteases that can activate and sensitise PAR2 expressing-sensory neurons (201). Injecting this supernatant (without cancer cells) in mice results in a severe and protracted mechanical allodynia. Analgesic approaches like serine protease blockade or mast cell depletion eliminate or lessen this nociceptive effect, respectively. Moreover, PAR2 knockout mice do not display nociceptive behaviours following exposure to SCC cell supernatant. Patients with oral cancer may develop mechanical allodynia due to the constant production of serine proteases from malignant as well as non-malignant cells within the tumour microenvironment. In addition, activating PAR2 sensitises TRPV1 and TRPV4 receptors on nociceptive afferents causing mechanical allodynia and thermal hyperalgesia, respectively (202).

## Pharmacological interventions to treat cancer pain

The efficacy of pain management in cancer is limited by the multidimensional aspects of the pathophysiology of cancer, such as widespread localisation of metastases, skeletal-related events (SREs) or due to severe systemic side effects of chemotherapy treatment (203). Gabapentinoids and antidepressants are common first and second line treatments for treatment of neuropathic pain syndromes and indicated also for cancer pain (204), but drugs that act on peripheral tissues would be preferable due to mechanisms involved in the initiation of pain and primary afferent sensitisation. The added attractive benefit of targeting the peripheral nervous system in cancer pain states is the obvious circumvention of central side effects. Here we provide an overview of some conventional treatments and potential new targets for treating cancer pain states.

### Opioids

Opioids remain the principal treatment option for intractable malignant pain by acting on peripheral and central nervous system sites, i.e., through inhibition of presynaptic release of neurotransmitters from primary afferent terminals to induce postsynaptic hyperpolarisation of interneurons in the dorsal horn, as well as engaging top down opioidergic controls from higher centres (205, 206). Strong immediate-release opioids are recommended as rescue medication for episodes of breakthrough pain. However, preclinical studies suggest that morphine is less effective in treating bone cancer pain compared to other painful conditions (41, 47), possibly due to decreased expression of μ-opioid receptors (MORs) in both DRG and superficial dorsal horn neurons with disease progression (207, 208). Moreover, opioid treatment in CIBP may also be linked to disease progression, as observed in mice administered morphine that demonstrated accelerated sarcoma-induced bone pain, bone loss and fractures (45). Altered expression of MORs in CIBP can also be reversed with anti-NGF in rats (209).

### Agents targeting bone resorption

SREs such as pathological fractures, hypercalcemia of malignancy, and bone marrow failure/leukoerythroblastic anaemia, among others, are common complications of bone metastases. The occurrence of SREs in CIBP provides the rationale for bone targeting agents to manage CIBP and to reduce the occurrence of SREs by reducing bone resorption (210). Besides bone cancer, other painful syndromes such as osteoporosis and fracture repair are also associated with increased bone resorption (37). Bisphosphonates and Denosumab are osteoclast targeting molecules that inhibit bone resorption. Bisphosphonates have a phosphorus-carbon-phosphorus that enable resistance to hydrolysis; nitrogen-containing bisphosphonates prevent prenylation of small guanosine triphosphate binding proteins that are essential for osteoclast function and survival (211), and non-nitrogen containing bisphosphonates are metabolised as ATP analogues to induce osteoclast apoptosis (212). The human monoclonal antibody Denosumab targets RANKL to prevent the development, activation and survival of osteoclasts (213). Moreover, IL-6 and TNF-α can induce osteoclastogenesis and bone erosion through a non-canonical pathway, which is independent of the activation of RANK (214).

### Anti-NGF

In animal models of CIBP, administration of anti-NGF neutralising antibodies dramatically reduces cutaneous and skeletal pain (215–217) by preventing ectopic sprouting (150) and reducing loss of bone integrity associated with CIBP (218). In non-cancer skeletal pain models, anti-NGF therapy has shown promising results, e.g., in mice with femoral fracture, anti-NGF therapy reduces pain behaviours without affecting bone healing (219). In a murine model of autoimmune arthritis, anti-NGF neutralising antibodies reduce hyperalgesia and cachexia with no effect on joint destruction and gross inflammation (220). Anti-NGF therapy may reduce hyperalgesia through the re-establishment of homeostatic MOR expression at the level of DRG and dorsal horn, where its anti-nociceptive effects can be reversed with naloxone pre-treatment (209). In humans, several monoclonal antibodies that bind NGF (including tanezumab, fulranumab, and fasinumab) have been used in clinical studies in a range of chronic pain conditions such as osteoarthritis [for a comprehensive review, see (221)]. A phase 2 trial investigating the analgesic efficacy of tanezumab as add-on therapy to opioid medication in patients with metastatic bone pain has recently been completed, and previous randomised control trials showed that tanezumab was shown to have greater analgesic efficacy in patients with lower baseline opioid use and/or higher baseline pain (222). Although tanezumab has been tested as a potential analgesic for patients with osteoarthritis, it did not secure FDA approval due to safety concerns linking use of NGF inhibitors to accelerated joint damage, and publication of NICE guidelines for the use of tanezumab to treat moderate-to-severe osteoarthritis pain is currently suspended.

### Endothelins

The family of endothelins (ET) consists of ET-1, ET-2 and ET-3 peptides that act on ETA and ETB G-protein-coupled receptors (223, 224). ET-1 increases intracellular calcium in peripheral sensory neurons and activates PKC-ε, leading to phosphorylation and activation of TRPV1 channels expressed on nociceptive C-fibres (225). In addition to activating TRPV1 channels, ET-1 also modulates the activity of TTX-resistant sodium channels (226), likely through PKC (227). ET-1 increases the release of neuropeptides and glutamate from isolated sensory neurons through an ETA-dependent increase in intracellular calcium (228, 229). ET-1 also modulates N and L-type calcium channels in a biphasic manner; initially, ET-1 slows down the activity of these calcium channels and following that causes long-lasting facilitation (230). The effect of ET-1 on the membrane potential is also biphasic as ET-1 initially causes depolarisation along with non-selective inward cationic currents (mostly calcium-mediated) followed by hyperpolarisation (probably as a result of calcium-activated outward potassium currents) (231). Furthermore, the release of calcium ions from intracellular stores contributes to the ET-1-mediated increase of intra-neuronal calcium ions concentration (229, 232). ETB receptors are primarily expressed in DRG satellite cells and ensheathing Schwann cells (233), where it triggers the production and release of PGE_2_ (234). ETB receptors also enhance the release of β-endorphin from keratinocytes and accordingly generate a local analgesia (235). ETA receptor activation leads to excitation of small-to-medium diameter DRG neurons (233), partly through the ability of ET-1 to activate voltage-gated sodium channels (226). According to (226), ET-1 predominantly activates TTX-resistant sodium channels on nociceptors by enabling these channels to open at more negative membrane potential. *In vivo* injection of ET-1 close to nerves enhances their excitability. In contrast, TTX-sensitive sodium channels are not affected by ET-1 (226). In addition, ET-1 represses currents generated by the outward delayed rectifier potassium channels in the vast majority of sensory neurons (236). ET-1-mediated suppression of outward delayed rectifier potassium currents combined with the activation shift that ET-1 causes on TTX-resistant sodium current greatly enhances the excitability of neurons (236). Besides the mechanisms mentioned above, ET-1 is of particular interest in CIBP for its ability to activate osteoblasts. In turn, osteoblasts can release RANKL, which can cause the activation and differentiation of pre-osteoclasts into mature osteoclasts leading to substantial bone degradation and acidosis-mediated pain (237) (see Figure 1). Due to its strong involvement in CIBP, ET-1 was tested as a potential target in preclinical models of CIBP. Another study showed that mice injected with 2472 sarcoma cell lines possess an increased level of ET-1 in the plasma compared to the controls (48). In the same study, antagonising ETA receptors reduced pain-associated behaviour in cancer-bearing mice, including both spontaneous and movement-evoked pain; in contrast, antagonising ETB receptors exacerbated pain-like behaviour in the CIBP mice. Similar promising results were obtained when ETA receptors were targeted in further preclinical investigation CIBP mechanisms (56).

### Other excitatory mediators

ATP is readily generated during inflammation and is abundantly expressed in malignant tissues (238). ATP receptors are known as purinergic receptors; P2Y receptors are members of the GPCR superfamily and P2X receptors are ligand-gated ion channels (239). Under physiological settings, it was discovered that cutaneous nerves have high concentrations of P2X3, but the periosteum and mineralised bone are nearly devoid of P2X3 (240). In line with these findings, it was demonstrated in a mouse model of CIBP (characterised by significant skin and skeletal hypersensitivity) that blocking P2X3 receptors with a monoclonal antibody reduces skin hypersensitivity (as measured by the von Frey test) while leaving skeletal pain-like behaviours largely unaffected (215). Another study reported a 5-fold increase in the expression of P2X3 in CGRP-positive epidermal nerve fibres in mice with osteolytic fibrosarcoma compared to control animals (241). Similarly, it was shown that the DRG neurons that innervate the rat tibia express P2X3 *de novo* in rat models of CIBP and systemic administration of the P2X2/3 antagonist AF-353 alleviates mechanical hypersensitivity without affecting cancer-induced bone deterioration. Not only does ATP contribute to pain peripherally, but it also has central effects demonstrated by *in vivo* recordings of dorsal horn neuronal excitability where intrathecal AF-353 decreases evoked action potential firing in significantly in a dose-dependent manner (144). For chemotherapy-induced neuropathy, preclinical studies suggest that glutamate can serve a protective function against neurotoxicity caused by cisplatin or paclitaxel, as measured by a reduction in proprioceptive loss and compromised performance in the rotarod test during dark cycles of rodents (82). Other protective strategies for cisplatin-induced neuropathy include ORG 2766 (93) and the recombinant human glial growth factor 2 (242).

## Conclusion

Cancer pain arising from disease pathology and/or cancer treatments will increase in prevalence with increasing and prolonged survival rates. Preclinical rodent models of cancer-induced bone pain and of chemotherapy induced neuropathy address prevalent chronic pain syndromes that are experienced by patients with bone metastases or undergoing chemotherapy treatments. Cancer pain is a complex condition driven by inflammatory, neuropathic and cancer-specific mechanisms. Peripheral cross talk between tumour cells, non-neuronal cells and neurons is a key process for the induction and maintenance of cancer pain states. Changes in the tumour microenvironment, such as bone remodelling and acidosis also contribute to the sensitisation of peripheral sensory neurons. Conventional pain management still relies on opioids and standard conventional first- and second-line analgesics that are normally indicated for neuropathic pain states. Improved understanding of mechanisms specific to cancer pain states is necessary to highlight new targets for pain relief. Modelling the heterogeneity of sensory dysfunction across different types of cancer pain is one of the biggest challenges in preclinical investigations. Future studies that use animal models of distinct cancer pain states to investigate the peripheral crosstalk between neuronal and non-neuronal cells will provide valuable insight into pathophysiological mechanisms of cancer pain.
